# From BIG Data Center to China National Center for Bioinformation

**DOI:** 10.1016/j.gpb.2023.10.001

**Published:** 2023-10-12

**Authors:** Yiming Bao, Yongbiao Xue

**Affiliations:** 1 National Genomics Data Center, Beijing Institute of Genomics, Chinese Academy of Sciences and China National Center for Bioinformation, Beijing 100101, China; 2 University of Chinese Academy of Sciences, Beijing 100049, China

## Background

### Big data challenges

In the late 1980s and early 1990s, three major international biological data centers were created: the DNA Database of Japan (DDBJ) [1], the European Bioinformatics Institute (EMBL-EBI) in the United Kingdom (UK) [2], and the National Center for Biotechnology Information (NCBI) in the United States (US) [3]. These three centers form the International Nucleotide Sequence Database Collaboration (INSDC) [4], which establishes international data standards and exchanges data among nucleotide sequence databases, namely, DDBJ, the European Nucleotide Archive [5], and GenBank [6]. For journal articles involving data, the convention is that the data need to be deposited into an internationally recognized database. For nucleotide sequences, the default database is usually one in INSDC. This situation imposes some challenges in China. Over the last decade or two, tremendous amount of data had been generated from large-scale national research projects based on genome sequencing. So had been the number of publications. Given the lack of internationally recognized data repositories in China, researchers in China relied heavily on databases like those from INSDC for both data archiving and retrieving. It is estimated that approximately 20% of the data and users of NCBI are from China, largely comparable to those from the US. However, technical issues such as slow internet connections and language barriers make data submission and acquisition very difficult.

### BIG Data Center

To address the big data-related challenges, Beijing Institute of Genomics (BIG), Chinese Academy of Sciences (CAS) launched BIG Data Center (BIGD) on February 29, 2016 [7]. The goal of BIGD is to advance life and health sciences by providing open access to a suite of resources, with the aim of translating big data into big discoveries and supporting worldwide activities in both academia and industry.

### National Genomics Data Center

In recent years, several policies related to scientific data have been released in China. Notably, the “Measures for Managing Scientific Data” was issued by the General Office of the State Council on March 17, 2018 (https://www.gov.cn/zhengce/content/2018-04/02/content_5279272.htm). Its key element is to promote the mandatory archiving of scientific data generated by national science and technology research projects in scientific data centers. The duties of these centers include (1) undertaking the integration and exchange of scientific data in relevant fields; (2) taking responsibility for the grading, categorization, processing, and analysis of scientific data; (3) ensuring the safety of scientific data and promoting the open sharing of scientific data in accordance with laws and regulations; and (4) strengthening scientific data exchanges and cooperation both domestically and internationally. Soon after that, on June 5, 2019, the Ministry of Science and Technology and Ministry of Finance of China announced the establishment of the first 20 National Scientific Data Centers. Among them, National Genomics Data Center (NGDC) [8] was created at BIG, in collaboration with two other CAS institutions, *i.e.*, the Institute of Biophysics and the Shanghai Institute of Nutrition and Health. NGDC is a national platform for archiving, managing, and processing a wide range of genomics-related data.

### China National Center for Bioinformation

With the solid foundation built up by BIGD and NGDC, and a great effort by CAS, the State Commission of Public Sectors Reform officially approved the formation of China National Center for Bioinformation (CNCB) on November 13, 2019 [9]. CNCB is affiliated with BIG, and is charged to undertake bioinformation data archiving, storage, management, and sharing, to perform frontier bioinformatics research, and to achieve translation and application.

## Progresses of CNCB

**Figure 1:**
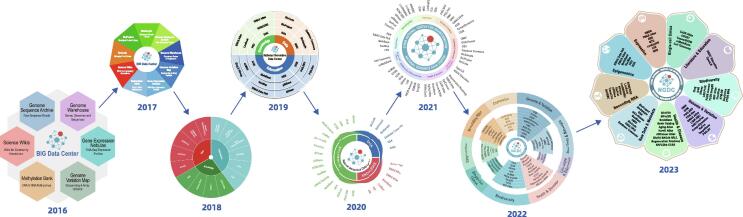
**Major resources of CNCB-NGDC from 2016 to 2023** During the past few years, the capacity of CNCB-NGDC had been growing from the initial 6 modules to a comprehensive multi-omics center with more than 70 database resources and tools in several directions.

During the past few years, the capacity of CNCB-NGDC has been growing from the initial 6 modules to a comprehensive multi-omics center with 71 database resources in several directions (Figure 1, https://ngdc.cncb.ac.cn) [10]. As of September 2023, CNCB-NGDC has more than 50 staff and 60 graduate students. It frequently upgrades infrastructure capabilities, currently with 1.6 gigabits per second (Gbps) of network bandwidth, 8300 computing cores, 266 teraflops (TFLOPs) of computing resources, and nearly 39 petabytes (PB) of storage. CNCB-NGDC follows largely the data structure, standards, and quality control processes of the corresponding databases in INSDC. In some cases, extra efforts such as auto-validation tools are made to compensate for the lack of standards in source data. CNCB-NGDC is listed by the journal *Nucleic Acids Research* (NAR) as one of the major database providers, together with EBI and NCBI [11].

### Basic multi-omics and meta data

CNCB-NGDC provides free, fast, and convenient multi-omics data submission and archive services for users from China and abroad. These include the BioProject and BioSample databases for omics research projects and samples, Genome Sequence Archive (GSA) family for raw sequence data [12], Genome Warehouse (GWH) for completed genomes [13], and GenBase (https://ngdc.cncb.ac.cn/genbase/) for virus and organelle genomes, as well as gene segments of all organisms, Gene Expression Nebulas (GEN) for transcriptomic profiles [14], Genome Variation Map (GVM) [15], and MethBank for whole-genome DNA methylation [16].

These databases start to play an increasing role in helping researchers, especially those in China, for data archiving and retrieving. As of December 31, 2022, GSA has reached 19 PB of omics data, submitted by 3519 users of 756 institutions from 19 countries/regions, covering more than 10 thousand research projects funded by various agencies. GSA-Human, a component of GSA, was designated in July 2022 as the backup platform for human genetic resource information by the Ministry of Science and Technology in China. Data submissions have been reported in over 2000 articles covering 475 journals. GSA has been recognized by major publishers, including Elsevier, Taylor & Francis, and Wiley. In particular, GSA is one of the mandatory data repositories for DNA sequence, RNA sequence, and genome assembly data by Springer Nature, the only such database apart from INSDC.

### Featured resources

CNCB-NGDC has developed a series of knowledgebases such as GWAS Atlas, EWAS Atlas, and TWAS Atlas for association studies with different omics types; resources for non-coding RNAs, including LncBook and LncRNAWiki; resources for important organisms, such as rice, soybeans, dogs, and sheep; a global catalog of biological databases “Database Commons” [17], which serves as a registration portal for databases published in the NAR Database Issue; and a suite of resources for health and diseases, *e.g.*, Aging Atlas, ASCancer Atlas, BrainBase, and RCoV19.

For example, RCoV19 is a resource for the 2019 novel coronavirus [18], which provides several functional modules on severe acute respiratory syndrome coronavirus 2 (SARS-CoV-2) genome sequences, genomic mutations, variant monitoring, early-warning of high-risk variants, online data analysis toolkits, and related literature. Serving more than 2.5 million visitors from 181 countries/regions worldwide, with total downloads of over 10 billion sequences, RCoV19 played an important role in the study of SARS-CoV-2. It provides scientific support for China-WHO joint research on the origins of SARS-CoV-2.

### Literature and tools

Literature services such as PubMed and bioinformatics tools represented by BLAST are essential parts of NCBI, besides omics databases. In line with these findings, CNCB-NGDC is developing the Open Library of Bioscience (OpenLB, https://ngdc.cncb.ac.cn/openlb/home), which provides open access to massive literature with links to relevant resources in CNCB-NGDC. Currently, it contains ∼ 36 million publications from PubMed, bioRxiv, and medRxiv. The BLAST (https://ngdc.cncb.ac.cn/blast/) service is also established at CNCB-NGDC. In addition to the commonly used nt and nr databases in NCBI’s BLAST service, some customized BLAST databases (*e.g.*, those for soybean genomes and Protist 10K Genomes Project Genes) are built in response to specific requests from users.

### Global collaborations

CNCB-NGDC partners with 44 popular databases in China (https://ngdc.cncb.ac.cn/partners) by integrating them into the BIG Search system (https://www.ngdc.cncb.ac.cn/search/). This search engine also combines search results from databases of NCBI and EBI through application programming interface (API). CNCB has been in close contact with INSDC in an effort to be part of it. With the help of NCBI, the GenBase database has integrated all GenBank records and provides web access to search, display, and download these records. A data sharing mechanism is also established to smoothly transfer public sequences deposited in GenBase to GenBank. Similar data exchange is also ongoing between the GSA and Sequence Read Archive (SRA) databases.

To promote biodiversity and health big data sharing in the world, CNCB together with Pakistan, Russia, Saudi Arabia, and Thailand launched the Open Biodiversity and Health Big Data (BHBD, https://ngdc.cncb.ac.cn/bhbd-alliance/) Alliance under the framework of the International Union of Biological Sciences. BHBD organized numerous conferences and trainings for researchers from its 28 members or 12 countries, published dozens of joint research papers, and secured multiple international collaboration research projects with a total funding of approximately 1 million US dollars.

## Future directions

Although CNCB has made itself into the top division of global biological data centers, there is still a long way to go to further enhance its capabilities. To cope with the exponential growth of data, an intelligent and automated system together with a professional curation team is urgently needed to efficiently manage the complete cycle involving data submission, release, sharing, and updating. The integration of all available data, powered by an advanced search engine, is necessary to make full use of multi-omics data. New algorithms, software packages, and tools are to be developed, and the existing heavily-used tools such as BLAST and University of California Santa Cruz Genome Browser will be provided and maintained. Cloud-based data storage coupled with tools and pipelines is inevitable, which can avoid the need to download data and greatly facilitate data analyses. Opportunities brought by novel artificial intelligence technologies such as AlphaFold 2 and ChatGPT have to be evaluated and utilized. Collaborations will be expanded to strengthen both domestic and global networks. Incentive benefit sharing mechanisms are to be investigated to promote data sharing [19].

## Competing interests

Both authors have declared no competing interests.

## CRediT authorship contribution statement

**Yiming Bao:** Conceptualization, Writing – original draft, Funding acquisition, Supervision. **Yongbiao Xue:** Conceptualization, Writing – review & editing, Supervision. Both authors have read and approved the final manuscript.
